# Supplementary material for: Phenotypic and Molecular-Phylogenetic Analyses Reveal Distinct Features of Crown Gall-Associated Xanthomonas Strains
Source: Microbiol Spectr. 2022 Feb 2;10(1):e00577-21. doi: 10.1128/spectrum.00577-21 (PMC8809331; doi:10.1128/spectrum.00577-21)
Supplement: SUPPLEMENTAL FILE 1 — Supplemental material. Download SPECTRUM00577-21_Supp_1_seq7.pdf, PDF file, 4.9 MB [file spectrum00577-21_supp_1_seq7.pdf]

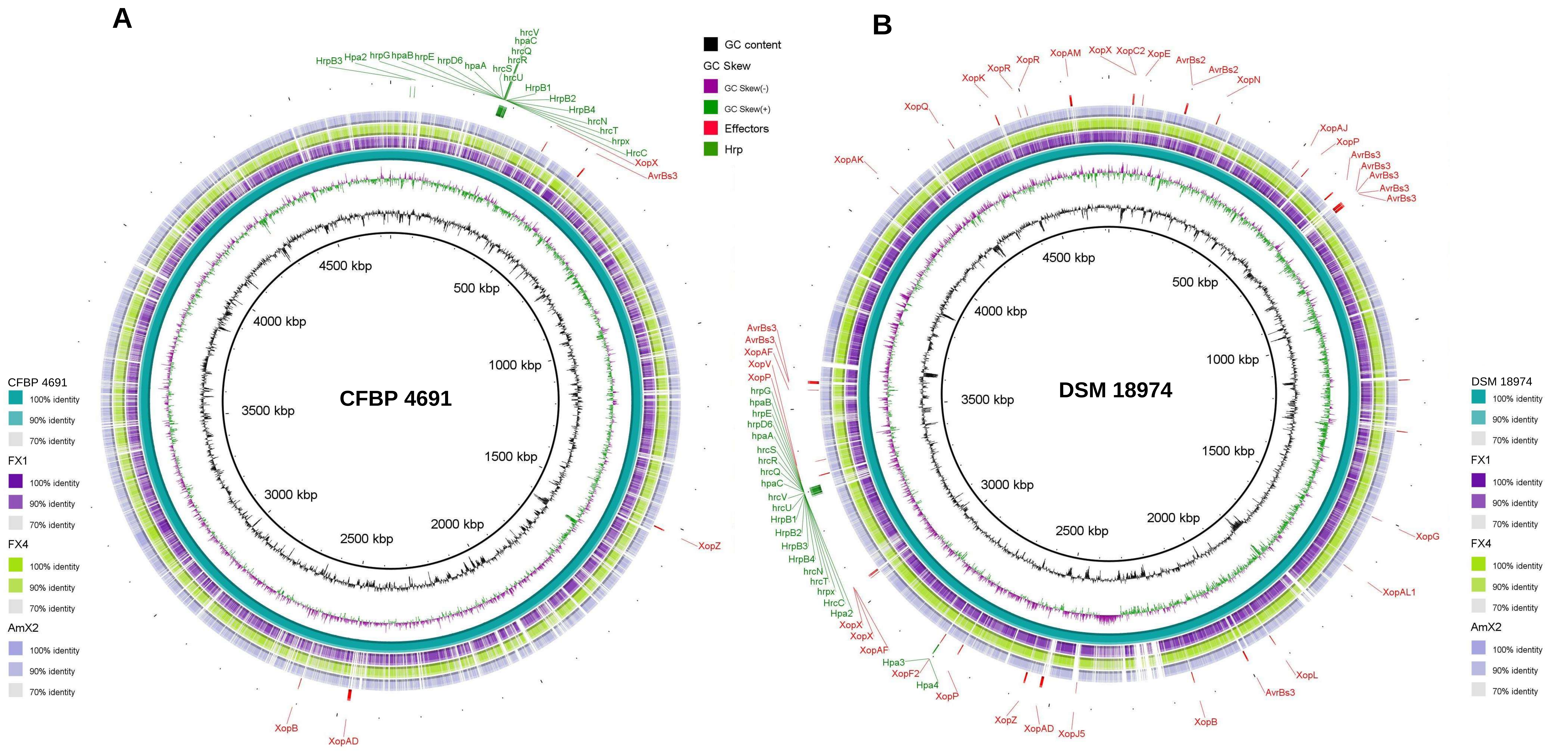


Figure S1: Pairwise genome collinearity alignment of the Xanthomonas strains sequenced in this study performed using BRIG 0.95. The genomes of X. theicola CFBP 4691 (A) and X. translucens pv. translucens DSM 18974 (B) were used as reference for the three strains FX1, FX4, and AmX2. Circular mapping against the reference genomes confirmed the absence of a large portion of the T3SS and Xops in the three sequences obtained in this study.

Figure S2: Visualization of genomic islands in the whole genome sequences of the three Xanthomonas strains sequenced in this study against the genomes of two reference strains X. theicola CFBP 4691 and X. translucens pv. translucens DSM 18974.

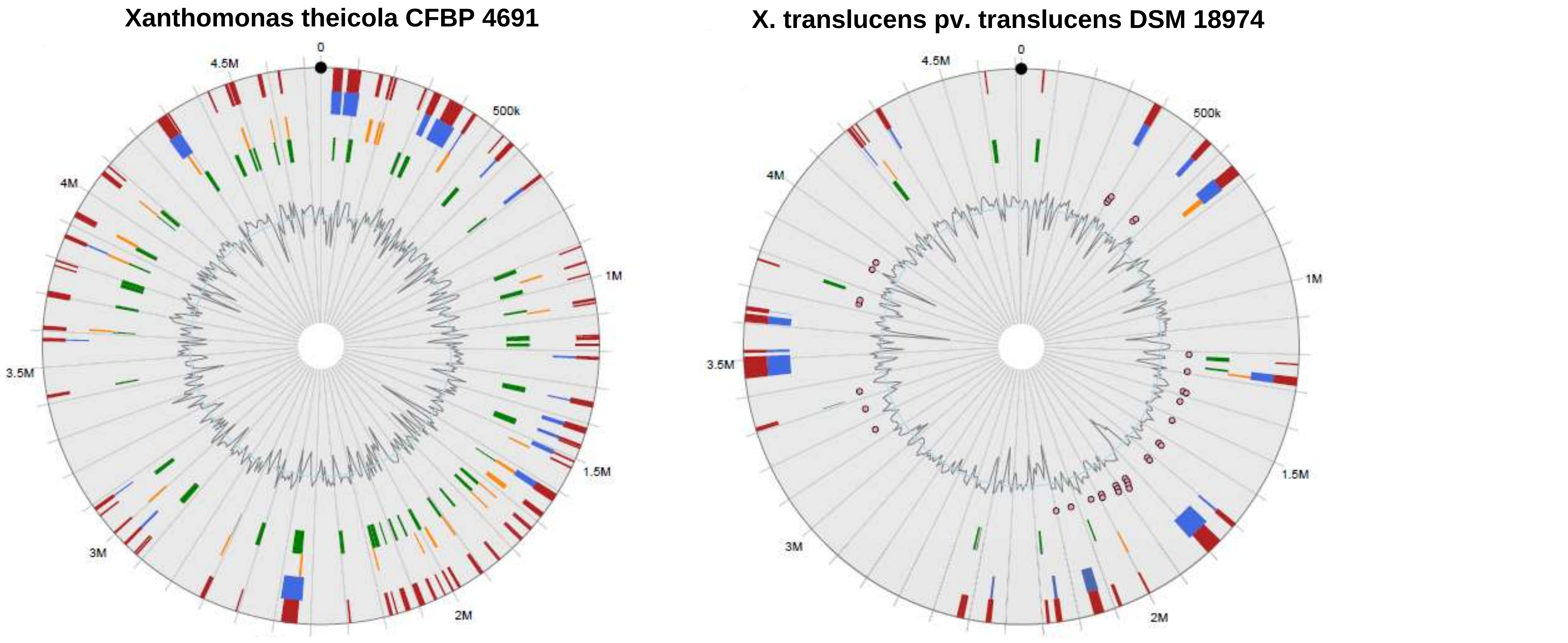


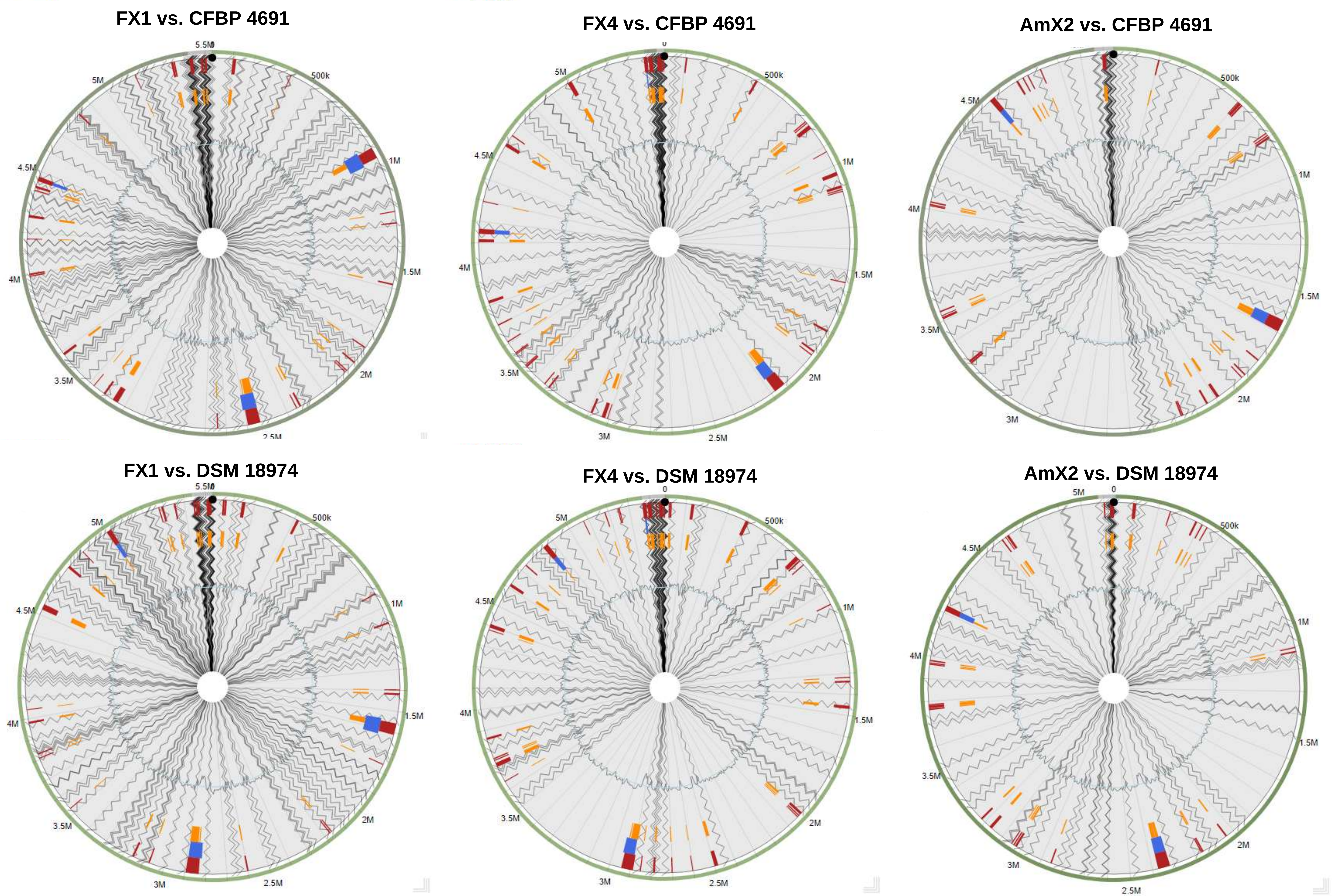

Table S1: Primer pairs used in this study.

| Prime name | 5´- 3´ sequence | Size of amplicon | Ann. Temp. | Target species/region | Reference |
|---|---|---|---|---|---|
| Xc-lip-F2 | TATGTGATGGTGCCGACCATTC | 777 | 57 | *Xanthomonas sp.* | Lee et al. 2009 |
| Xc-lip-R2 | GGACTTCGCGGTCCACGTCGTAGC | | | | |
| T1 | CCGCCATAGGGCGGAGCACCCCGAT | 139 | 53 | *Xanthomonas translucens* | Maes et al. 1996 |
| T2 | GCAGGTGCGACGTTTGCAGAGGGATCTTCTGCAAA | | | | |
| Bs-XeF | CATGAAGAACTCGGCGTATCG | 173 | 64 | *X. euvesicatoria* pv. | Koenraadt et al. 2009 |
| Bs-XeR | GTCGGACATAGTGGACACATAC | | | euvesicatoria | |
| Bs-XvF | CCATGTGCCGTTGAAATACTTG | 138 | 64 | *X. vesicatoria* | Koenraadt et al. 2009 |
| Bs-XvR | ACAAGAGATGTTGCTATGATTTGC | | | | |
| Bs-XgF | TCAGTGCTTAGTTCCTCATTGTC | 154 | 64 | *X. hortorum pv. gardnri* | Koenraadt et al. 2009 |
| Bs-XgR | TGACCGATAAAGACTGCGAAAG | | | | |
| Bs-XpF | GTCGTGTTGATGGAGCGTTC | 197 | 64 | *X. euvesicatoria* pv. | Koenraadt et al. 2009 |
| Bs-XpR | GTGCGAGTCAATTATCAGAATGTGG | | | *perforans* | |
| X-gyrB1F | ACGAGTACAACCCGGACAA | 904 | 57 | *gyrB* | Young et al. 2008 |
| X-gyrB1R | CCCATCARGGTGCTGAAGAT | | | | |
| P-X-ATPD-F | GGGCAAGATCGTTCAGAT | 868 | 60 | *atpD* | Boudon et al. 2005 |
| P-X-ATPD-R | GCTCTTGGTCGAGGTGAT | | | | |
| P-X-EFP-F | TCATCACCGAGACCGAATA | 445 | 62 | *efp* | Boudon et al. 2005 |
| P-X-EFP-R | TCCTGGTTGACGAACAGC | | | | |
| XtrpodF | TGGAACAGGGCTATCTGACC | 674 | 54 | *rpoD* | Young et al. 2008 |
| XtrpodR | CATTCYAGGTTGGTCTGRTT | | | | |

**A**

| Strain | Size | BUSCO notation assessment results | | | | |
|---|---|---|---|---|---|---|
| AmX2 | 5,071 kbp | C:99.8% | [D:17%], | F:0.09%, | M:0.09%, | n:1152 |
| FX1 | 5,387 kbp | C:99.6% | [D:17%], | F:0.26%, | M:0.09%, | n:1152 |
| FX4 | 5,383 kbp | C:99.9% | [D:17%], | F:0.0%, | M:0.09%, | n:1152 |
| DSM 18974 | 4,715 kbp | C:99.8% | [D:0%], | F:0.0%, | M:0.17%, | n:1152 |

C: Complete; D: Duplicated; F: Fragmented; M: Missing; n: No. of genes

**B**

| *ooxA* | AmX2 | FX1 | FX4 | *P. kilonensis* |
|---|---|---|---|---|
| Identity | 27.1 | 25.8 | 25.8 | 35.8 and 29.3 |
| Query coverage | 73 | 66 | 66 | 85 and 84 |
| Contig No. | 5 | 15 | 1 | 17 and 23 |
| | | | | |
| *ooxB* | AmX2 | FX1 | FX4 | |
| Identity | 23.5 | 26 and 23 | 26 and 23 | 41.5 and 25.6 |
| Query coverage | 64 | 39 and 90 | 39 and 90 | 95 and 98 |
| Contig No. | 5 | 15 and 5 | 1 and 21 | 17 and 23 |
| *ooxAB* | AmX2 | FX1 | FX4 | |
| Identity | 25 | 25 | 25 | 35.8 and 29.3 |
| Query coverage | 66 | 66 | 66 | 85 and 84 |
| Contig No. | 1 | 15 | 1 | 17 and 23 |

**Table S2:** Quality assessment of genome assembly and annotation using BUSCO online service confirmed the accuracy and completeness of all three genomes compared to the complete genome sequence of *X. translucens* DSM 18974 (A). BLAST-based in-silico analyses using the sequences of ooxA, ooxB, and ooxAB genes against the three genome sequences obtained in this study revealed the presence of these genes in the three strains (B).

**Table S3:** Genomic features of the gall-associated *Xanthomonas* strains sequenced in this study in comparison to the phylogenetically closely related reference strains.

| Taxon | Strain | Host | **Genome Information** | | | | | | **GenBank Accession Numbers** |
|---|---|---|---|---|---|---|---|---|---|
| | | | **Genome length (kbp)** | **G + C content (%)** | **No. of protein-coding genes** | **No. of RNAs** | **No. of pseudo-genes** | **Genome Coverage (x)** | |
| *Xanthomonas* sp. | FX1 | *Ficus benjamina* | 5,387 | 68.8 | 4,502 | 57 | 40 | 22 | JAFJNT000000000 |
| *Xanthomonas* sp. | FX4 | *Ficus benjamina* | 5,383 | 68.8 | 4,468 | 64 | 68 | 115 | JAFIWB000000000 |
| *Xanthomonas* sp. | AmX2 | *Amaranthus* sp. | 5,071 | 69.8 | 4,170 | 61 | 41 | 124 | JAFIWC000000000 |
| *X. translucens* pv. *graminis* | NCPPB 3709 | Grass | 4,187 | 68.8 | 3,337 | 59 | 233 | ND | NZ_FLUK01000000 |
| *X. translucens* pv. *undulosa* | ICMP 11055 | *Triticum aestivum L.* | 4,761 | 67.8 | 3,835 | 63 | 224 | 118 | CP009750 |
| *X. translucens* pv. *translucens* | UPB886 | *Hordeum vulgare* | 4,674 | 67.9 | 3,681 | 63 | 240 | 10 | NZ_VCIH01000000 |
| *X. translucens* pv. *translucens* | DSM 18974 | *Hordeum vulgare* | 4,715 | 67.7 | 3,804 | 64 | 190 | ND | LT604072 |
| *X. translucens* pv. *cerealis* | NCPPB 1943 | *Hordeum vulgare* | 4,410 | 67.3 | 3,583 | 58 | 358 | 197 | NZ_MADM00000000 |
| *X. translucens* pv. *arrhenatheri* | LMG 727 | *Arrhenatherum elatius* | 4,753 | 68.3 | 3,791 | 58 | 137 | ND | NZ_CXOI00000000.1 |
| *X. translucens* pv. *phlei* | LMG 730 | *Phleum pratense* | 4,396 | 68.4 | 3,545 | 58 | 212 | ND | NZ_CXOJ01000000 |
| *X. translucens* pv. *poae* | ATCC 33804 | *Poa trivialis* | 4,498 | 67.5 | 3,789 | 57 | 415 | 188 | NZ_MADN00000000 |
| *X. hyacinthi* | CFBP 1156 | *Hyacinthus orientalis* | 4,918 | 68.1 | 4,011 | 63 | 318 | 306 | CP043476 |
| *X. translucens* | 569 | Sorghum (*Sorghum bicolor)* | 5,323 | 69.7 | 4,414 | 60 | 52 | 282 | NZ_VIWM01000000 |
| *X. theicola* | CFBP 4691 | *Camellia sinensis* | 4,744 | 68.4 | 3,766 | 61 | 665 | 264 | CP049017 |
| *Xanthomonas* sp. | NCPPB 1128 | *Phaseolus vulgaris* | 4,950 | 68.9 | 4,133 | 66 | 55 | 80 | NZ_LFME00000000 |
| *X. sacchari* | NCPPB 4393 | Insect associated with banana plant | 4,898 | 69.0 | 4,168 | 65 | 163 | 80 | NZ_AGDB00000000 |
| *X. albilineans* | CFBP 2523 | *Saccharum officinarum* | 3,684 | 63.1 | 2,996 | 81 | 168 | 100 | NZ_MDCB00000000 |
| *X. sontii* | PPL1 | *Oryza sativa* | 4,820 | 69.0 | 3,951 | 58 | 198 | 78 | NQYO01000001 |

| Taxon | Strain | Accession number | No. of contigs | Assembly Method | Annotation Method | Annotation Software revision | Annotation Method |
|---|---|---|---|---|---|---|---|
| Xanthomonas sp. | FX1 | JAFJNT000000000 | 152 | SPAdes v. 3.12 | NCBI | 5.1 | Best-placed reference protein set-GeneMarkS-2+ |
| Xanthomonas sp. | FX4 | JAFIWB000000000 | 89 | SPAdes v. 3.12 | NCBI | 5.1 | Best-placed reference protein set-GeneMarkS-2+ |
| Xanthomonas sp. | AmX2 | JAFIWC000000000 | 75 | SPAdes v. 3.12 | NCBI | 5.1 | Best-placed reference protein set-GeneMarkS-2+ |
| *X. translucens* pv. *graminis* | NCPPB 3709 | NZ_FLUK01000000 | 300 | ND | NCBI RefSeq | 4.12 | Best-placed reference protein set-GeneMarkS-2+ |
| *X. translucens* pv. *undulosa* | ICMP11055 | CP009750 | 1 | HGAP3 v. SMRTAnalysis 2.2 | NCBI | 2.8 (rev. 449627) | Best-placed reference protein set; GeneMark-S+ |
| *X. translucens* pv. *translucens* | UPB886 | NZ_VCIH01000000 | 1 | HGAP v. 4 | NCBI RefSeq | 5.1 | Best-placed reference protein set-GeneMarkS-2+ |
| *X. translucens* pv. *translucens* | DSM 18974 | LT604072 | 1 | ND | ND | ND | ND |
| *X. translucens* pv. *cerealis* | NCPPB1943 | NZ_MADM00000000 | 251 | Velvet v. 1.0.15 | NCBI RefSeq | 4.12 | Best-placed reference protein set-GeneMarkS-2+ |
| *X. translucens* pv. *arrhenatheri* | LMG 727 | NZ_CXOI00000000.1 | 89 | ND | NCBI RefSeq | 4.12 | Best-placed reference protein set-GeneMarkS-2+ |
| *X. translucens* pv. *phlei* | LMG730 | NZ_CXOJ01000000 | 142 | ND | NCBI RefSeq | 4.12 | Best-placed reference protein set-GeneMarkS-2+ |
| *X. translucens* pv. *poae* | ATCC 33804 | NZ_MADN00000000 | 674 | Velvet v. 1.0.15 | NCBI RefSeq | 4.12 | Best-placed reference protein set-GeneMarkS-2+ |
| *X. hyacinthi* | CFBP 1156 | CP043476 | 1 | HGAP v. 4.0 | NCBI RefSeq | 4.9 | Best-placed reference protein set-GeneMarkS-2+ |
| *X. translucens* | 569 | NZ_VIWM01000000 | 16 | SPAdes v. 3.13.0 | NCBI RefSeq | 4.12 | Best-placed reference protein set-GeneMarkS-2+ |
| *X. theicola* | CFBP 4691 | CP049017 | 435 | HGAP v. 4.0 | NCBI RefSeq | 4.11 | Best-placed reference protein set-GeneMarkS-2+ |
| *Xanthomonas* sp. | NCPPB1128 | NZ_LFME00000000 | 71 | SPAdes v. 3.5.0 | NCBI RefSeq | 5.1 | Best-placed reference protein set-GeneMarkS-2+ |
| *X. sacchari* | NCPPB 4393 | NZ_AGDB00000000 | 470 | Velvet v. 1.1.03 | NCBI RefSeq | 5.0 | Best-placed reference protein set-GeneMarkS-2+ |
| *X. albilineans* | CFBP2523 | NZ_MDCB00000000 | 148 | Velvet v. 1.2.07; | NCBI RefSeq | 4.11 | Best-placed reference protein set-GeneMarkS-2+ |
| *X. sontii* | PPL1 | NQYO01000001 | 332 | CLC NGS Cell v. 10 | NCBI RefSeq | 4.2 | Best-placed reference protein set-GeneMarkS-2+ |
| *X. oryzae* pv. *oryzae* | PXO99A | NC_010717 | 1 | Celera Assembler v. 7.0; SVN revision | NCBI RefSeq | 5.0 | Best-placed reference protein set-GeneMarkS-2+ |

ND: Not determined

**Table S4:** Assembly and annotation information for the genome sequences of *Xanthomonas* strains used in this study.

**Table S5:** In silico screening for bacteriocins and antibiotic peptides among the clade-I xanthomonad genome sequences analyzed in this study.

| Taxon | Strain | Start codon | End codon | gene ID | Class |
|---|---|---|---|---|---|
| *Xanthomonas* sp. | FX1 | 29117 | 49117 | SU2U6DHN013 | Sactipeptides |
| | | 4098 | 24404 | SU2RDK4F016 | Zoocin_A |
| *Xanthomonas* sp. | FX4 | 272837 | 293143 | SU316SGK013 | Zoocin_A |
| *Xanthomonas* sp. | AmX2 | 1 | 16792 | SU25J9VB013 | Zoocin_A |
| | | 59555 | 79909 | SU2CS5MN013 | Rhodanodin |
| *X. translucens* pv. *graminis* | NCPPB 3709 | 6493 | 13507 | SU3F4TS1016 | Sactipeptides |
| | | 1 | 14668 | SU3FU8EM013 | Zoocin_A |
| *X. translucens* pv. *undulosa* | ICMP 11055 | 3175859 | 3196165 | SU3S1T6U016 | Zoocin_A |
| *X. translucens* pv. *translucens* | UPB886 | 1556565 | 1576871 | SU3WH7RE016 | Zoocin_A |
| *X. translucens* pv. *translucens* | DSM 18974 | 2853584 | 2873890 | SU8PDA47013 | Zoocin_A |
| *X. translucens* pv. *cerealis* | NCPPB 1943 | 14585 | 34870 | SU4ACKR701R | Zoocin_A |
| | | 428 | 20428 | SU497DWS01R | Sactipeptides |
| *X. translucens* pv. *arrhenatheri* | LMG 727 | 15980 | 36286 | SU4ACKR701R | Zoocin_A |
| | | 71606 | 91606 | SU4ACKR701R | Sactipeptides |
| *X. translucens* pv. *poae* | ATCC 33804 | -6589 | 13411 | SU5MG31H013 | Sactipeptides |
| | | 1522 | 18478 | SU5PF8HV01R | Microcin |
| | | 0 | 7536 | SU5PF8HV01R | Zoocin_A |
| | | 3934 | 16066 | SU5PF8HV01R | Sactipeptides |
| *X. hyacinthi* | CFBP 1156 | 3858063 | 3878369 | SU5WTNE1013 | Zoocin_A |
| *X. translucens* | 569 | 378461 | 398767 | SU61CZRB013 | Zoocin_A |
| *X. theicola* | CFBP 4691 | 35588 | 55588 | SU6F10G2016 | Sactipeptides |
| *Xanthomonas* sp. | NCPPB 1128 | 8891 | 28891 | SU6S1F3901R | Sactipeptides |
| | | 464 | 20770 | SU6TKGCX013 | Zoocin_A |
| | | 8197 | 11803 | SU6TKGCX013 | Sactipeptides |
| *X. sacchari* | NCPPB 4393 | 0 | 14755 | SU7CFRHR016 | Zoocin_A |
| | | 6854 | 26854 | SU7DUS55013 | Sactipeptides |
| *X. albilineans* | CFBP 2523 | 70001 | 90307 | SU7DUS55013 | Zoocin_A |
| | | 269267 | 289267 | SU7VAXPR01R | Lanthipeptide_class_IV |
| | | 303776 | 323776 | SU8H1X8S013 | Microcin |

**Table S6:** Prophages within the genome sequences of clade-I xanthomonad strains detected using the online service PHASTER.

| Taxon | Strain | Region Length (Kb) | Completeness | Total Protein | gene ID | Most Common Phage | GC % |
|---|---|---|---|---|---|---|---|
| *Xanthomonas* sp. | FX1 | 12.8 | incomplete | 12 | SXHAS80N013 | Escherichia phage 500465-1; NC_049342 | 68.9 |
| *Xanthomonas* sp. | FX4 | 12.8 | incomplete | 12 | SXHRWEJF01R | Escherichia phage 500465-1; NC_049342 | 68.9 |
| *Xanthomonas* sp. | AmX2 | 11 | incomplete | 10 | SXGGHMWK016 | Escherichia phage 500465-1; NC_049342 | 69.8 |
| | | 0.7 | incomplete | 11 | SXGB4WXJ013 | Ralstonia phage phiRSA1; NC_009382 | 63.7 |
| *X. translucens* pv. *graminis* | NCPPB 3709 | 6.2 | incomplete | 8 | SXJ587H6013 | Escherichia phage 500465-1; NC_049342 | 68.9 |
| *X. translucens* pv. *undulosa* | ICMP 11055 | 24.5 | questionable | 14 | SXJFM2NJ016 | Ralstonia phage phiRSA1; NC_009382 | 63.9 |
| | | 33.7 | intact | 15 | SXJED8BV013 | Shigella phage SfIV; NC_022749 | 65.0 |
| | | 13.5 | incomplete | 8 | SXJBD8KU013 | Stenotrophomonas phage phiSMA6 ; NC_043029 | 66.3 |
| | | 44.5 | intact | 67 | SXJ9R7KG013 | Pseudomonas phage phiPSA1; NC_024365 | 67.2 |
| *X. translucens* pv. *translucens* | UPB886 | 12.3 | incomplete | 10 | SXJP44SE013 | Paenibacillus phage Tripp; NC_028930 | 65.0 |
| | | 9.1 | incomplete | 8 | SXJRKK8B016 | Synechococcus phage ACG-2014f; NC_047714 | 65.3 |
| | | 10.6 | questionable | 10 | SXJT2AG6013 | Listeria phage LMTA-34; NC_042048 | 66.6 |
| *X. translucens* pv. *translucens* | DSM 18974 | 12.3 | incomplete | 10 | SXK3D14E013 | Paenibacillus phage Tripp; NC_028930 | 65.0 |
| *X. translucens* pv. *cerealis* | NCPPB 1943 | 6.1 | incomplete | 7 | SXK72C59016 | Agrobacterium phage Atu_ph07; NC_042013 | 63.2 |
| *X. translucens* pv. *arrhenatheri* | LMG 727 | 9.3 | incomplete | 9 | SXKGYC2N013 | Escherichia phage 500465-1; NC_049342 | 69.1 |
| *X. translucens* pv. *phlei* | LMG 730 | 5.2 | questionable | 6 | SXKKGADM016 | Coliphage phi-X174; NC_001422 | 44.8 |
| *X. translucens* pv. *poae* | ATCC 33804 | 7.3 | incomplete | 7 | SXKXT95Y013 | Escherichia phage PA28; NC_041935 | 67.0 |
| *X. hyacinthi* | CFBP 1156 | 11.7 | incomplete | 11 | SXM3AX8A013 | Escherichia phage 500465-1_NC_049342(6) | 68.9 |
| *X. translucens* | 569 | 12.7 | incomplete | 13 | SXMMNTAM013 | Escherichia phage 500465-1_NC_049342(6) | 69.6 |
| *X. theicola* | CFBP 4691 | 4.4 | incomplete | 6 | SXNNYR3W016 | Synechococcus phage ACG-2014f; NC_047712 | 67.2 |
| | | 24.5 | intact | 27 | SXNTYE2H013 | Xanthomonas phage Xp15; NC_007024 | 66.6 |
| *Xanthomonas* sp. | NCPPB 1128 | 88.3 | intact | 111 | SXPH7FS9013 | Xylella phage Xfas53; NC_013599 | 67.8 |
| *X. sacchari* | NCPPB 4393 | 7.6 | intact | 14 | SXPMZ2W4013 | Stenotrophomonas phage PSH1; NC_010429 | 62.4 |
| *X. albilineans* | CFBP 2523 | 5.2 | incomplete | 9 | SXR4BE1F013 | Shigella phage SfIV; NC_022749 | 61.6 |
| | | 9.6 | incomplete | 12 | SXR6S4D4013 | Escherichia phage 500465-1_NC_049342 | 63.2 |
| | | 21.9 | intact | 27 | SXR8XRZC013 | Salmonella phage 118970_sal3; NC_031940 | 61.8 |
| | | 6.7 | incomplete | 8 | SXRDR02G013 | Proteus phage Mydo; NC_048741 | 63.8 |
| | | 7.9 | intact | 15 | SXRG3EEA013 | Stenotrophomonas phage phiSMA6 ; NC_043029 | 57.8 |
| | | 8.5 | questionable | 18 | SXRMUVS5016 | Stx2-converting phage Stx2a_WGPS9;NC_049923 | 59.0 |

**Reference for supplementary material:**


Boudon, S., Manceau, C., & Nottéghem, J. L. (2005). Structure and origin of *Xanthomonas arboricola* pv. *pruni* populations causing bacterial spot of stone fruit trees in Western Europe. Journal of Phytopathology, 95(9), 1081-1088.

Koenraadt H, van Betteray B, Germain R, Hiddink G, Jones JB, Oosterhof J, 2009. Development of specific primers for the molecular detection of bacterial spot of pepper and tomato. In: Aysan Y, Saygili H, Sahin F, Proceedings of the second International Symposium on Tomato Diseases. October 8, 2007, Kusadasi, Turkey. International Society for Horticultural Science, Acta Horticulturae, 808, 99–102.

Lee YA, Sung AN, Liu TF, Lee YS (2009) Combination of chromogenic differential medium and estA-specific PCR for isolation and detection of phytopathogenic *Xanthomonas* spp. Appl Environ Microbiol 75: 6831-6838

Maes M, Garbeva P, Kamoen O. 1996. Recognition and detection in seed of the *Xanthomonas* pathogens that cause cereal leaf streak using rDNA spacer sequences and polymerase chain reaction. Phytopathology 86:63-69.

Young, J. M., Park, D. C., Shearman, H. M., & Fargier, E. (2008). A multilocus sequence analysis of the genus *Xanthomonas*. Systematic and applied microbiology, 31(5), 366-377.
